# Preoperative Diagnosis Failure for a Rare Gastric Collision Tumor: A Case Report

**DOI:** 10.3390/diagnostics11040633

**Published:** 2021-04-01

**Authors:** Rabie E. Elshaer, Eid R. Elgammal, Amr M. Elmistekawy, Walaa A. Ghannam, Ahmed E. Elshamy, Sally Y. Abed, Sawsan A. Zaitone

**Affiliations:** 1 Department of Pathology, Faculty of Medicine, Al-Azhar University, Cairo 11651, Egypt; ra88871@azhar.edu.eg; 2 Department of Surgery, Faculty of Medicine, Al-Azhar University, Cairo 11651, Egypt; elgammal.ead@gmail.com; 3 Department of Internal Medicine, Faculty of Medicine, Al-Azhar University, Cairo 11651, Egypt; drmistekawy@azhar.edu.eg; 4 Department of Pathology, Faculty of Medicine, Suez University, Suez 43533, Egypt; Walaa.ghanam@med.suezuni.edu.eg; 5 Department of Radiology, Faculty of Medicine, Al-Azhar University, New Damietta 71524, Egypt; ahmed.elbastawesy@azhar.edu.eg; 6 Department of Respiratory Care, College of Applied Medical Science in Jubail, Imam Abdulrahman Bin Faisal University, Jubail 35816, Saudi Arabia; Syabed@iau.edu.sa; 7 Department of Pharmacology & Toxicology, Faculty of Pharmacy, Suez Canal University, Ismailia 41522, Egypt; 8 Department of Pharmacology & Toxicology, Faculty of Pharmacy, University of Tabuk, Tabuk 47512, Saudi Arabia

**Keywords:** case report, GISTs, inflammatory florid polyp, gastric neoplasm

## Abstract

Gastrointestinal stromal tumors (GISTs) are common mesenchymal tumors of the gastrointestinal tract (GIT), usually occur as a solitary neoplasm. Inflammatory florid polyp (IFP) is a solitary rare benign lesion of the gastrointestinal tract, mainly occur in the gastric antrum, whose atypical presentation can mimic GISTs or other malignant tumors, therefore the synchronous occurrence of GISTs and IFP is extremely rare. We had a case of a 58-year-old man that was presented with recurrent epigastric pain and recurrent melena. Upper endoscopic examination revealed a large polypoid antrum polyp measured 7 cm at greatest dimension with focal ulceration. Clinical and radiological features did not reach the definite diagnosis until histopathological evaluation with immunohistochemical analysis was performed. Surgical intervention is recommended and partial gastrectomy was done with wide resection margins. Histological examination revealed two distinct GISTs and IFP parts presenting a collision tumor that showed spindle and epitheloid cells consistent with GISTs with histological features of florid polyp showed a characteristic perivascular onion-skin arrangement of spindle cells with dense chronic inflammatory infiltrate including eosinophils and lymphocytes. Immunohistochemical studies have been done and revealed an association between GISTs and IFP. To the best of our knowledge, this is the first case of a collision tumor consisting of a GIST and an IFP arising in the stomach. In conclusion, the gastrointestinal stromal tumor is the comments mesenchymal tumor of GIT and IFP is a rare benign lesion of GIT therefore association between GIST and IFP as a collision tumor is extremely rare.

## 1. Introduction

Collision tumor is defined as the occurrence of two adjacent but histologically different tumors without admixture between the two tumors at the interface area [1,2]. Gastrointestinal stromal tumors (GISTs) are the most common mesenchymal tumors of the gastrointestinal tract (GIT) [3]; they arise from the interstitial cells of Cajal within the myenteric plexus of the muscularis propria [4]. It can occur anywhere along GIT with the majority of them occur in the stomach (60–70%) followed by the small intestine (25–30%) and rarely in the colon or esophagus [4]. The exact etiology of GISTs is not yet known; most of the cases are sporadic and may be due to mutations in proto-oncogene KIT (exon 11) [5]. It usually occurs in the older adult and very rarely in children and young. The most common presentation is gastrointestinal bleeding or abdominal pain. Histologically, GISTs are classified into spindle type, epithelioid type, or mixed type [6]. Most of them are positive for (cluster of differentiation 117) CD117, (discovered on GIST-1) DOG-1, and (cluster of differentiation 34) CD34 [7]. Inflammatory florid polyps (IFPs) are extremely rare reactive lesions that arise within the submucosa of the GIT, and represent less than 0.1% of all gastric polyps [8]. Histopathologically, GISTs usually take place within the submucosa but a mucosal extension can occur in some cases. The lesion may be hypocellular or hypercellular with variation in the degree of vascularity and number of eosinophils [9]. However, the characteristic feature of perivascular onion skinning was present in most of the cases, and the majority of IFPs express CD34 marker [10]. Association between GISTs and IFPs is very rare and to the best of our knowledge, it has not been reported earlier.

## 2. Material and Methods

### 2.1. Preoperative Findings

#### 2.1.1. Clinical Data

A 58-year-old Egyptian male was presented with abdominal epigastric pain and intermittent melena with gradual onset and progressive course and then became persistent with severe anemia. He was H. pylori positive and preoperative hemoglobin (Hb) was 10.6 g/dL and mean corpuscular volume (MCV) value was 81.3 FL. 

#### 2.1.2. Medical History

The patient complained of abdominal discomfort and epigastric pain for 6 months, then he got intermittent hematemesis and melena 2 weeks before endoscopic examination.

#### 2.1.3. CT Enterocolongraphy

Radiological assessment was performed on a multi-detector computed tomography (MDCT) scanner after proper preparation of the patient was done through fasting 4–6 h before the examination, then the patient drank 1.5 L of oral contrast (mannitol) over 30–60 min, then intravenous contrast was injected.

### 2.2. Endoscopic Examination

Endoscopic examination (gastroscopy) was done using the Olympus-240 Gastroscope (EVIS 240, Olympus Optical Co., Ltd., Tokyo, Japan) after verbal and written consent, under conscious sedation (midazolam 4 mg, i.v.) while the patient on his left lateral position, and the vital signs were continuously monitored throughout the entire procedure. The screening examination was done throughout the esophagus, stomach down to the second part of the duodenum.

### 2.3. Clinical Assessment and Intra-Operative Findings

Under general anesthesia, an upper middle incision was done. Exploration of abdominal metastasis in the liver, abdominal lymph nodes, and para-aortic lymph nodes was performed. Open examination of the stomach was also performed. Distal gastrectomy with antro-gastric anastomosis was done manually without stapling maneuver. Further, complete hemostasis with introducing corrugating rubber drain was done. Devascularization of the greater curvature until short gastric vessels with preservation of right gastric vessels, excision of the body of the stomach with the mass then reconstruction of the stomach by Billroth I operation.

### 2.4. Operative Findings 

#### Gross Findings

A partial gastrectomy for the specimen was performed with an omentum piece. The stomach measurements were determined and then the stomach was sectioned for the examination of the mucosa and any focal erosions. Further, we looked for the presence of any lymph nodes.

### 2.5. Histopathology Examination

Sections from the dissected mass were fixed in 10% paraformaldehyde solution for 24 h and then impeded in paraffin wax to prepare paraffin blocks. Then, 4 μm sections were deparaffinized, dehydrated, and processed for staining with hematoxylin and eosin for routine examination under a light microscope. Plastic growth and mucosal surfaces were examined, and any possible ulcerations were detected.

### 2.6. Immunohistochemical Assessment

Sections from the dissected polypoid mass were immunostained for [CD117 (c-KIT), DOG-1 (discovered on GIST1), CD34, smooth muscle actin (SMA), S100 and desmin] using fully automated autoimmunostainer using primary polyclonal antibodies (Anti Rabbit specific HRP/DAB (ABC) immunohistochemistry detection kit, ready to use) from (Dako, Glostrup, Denmark). The reaction was visualized using horseradish peroxidase immunohistochemistry detection kits (Dako, Denmark).

## 3. Results

### 3.1. CT Enterocolongraphy

**Figure 1:**
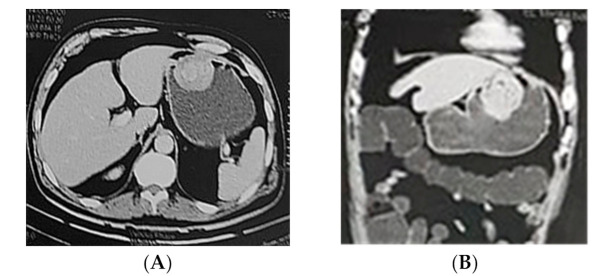
Axial (**A**) and coronal (**B**) CT enterocolography showing anterior gastric wall endophytic mass lesion. No outlet obstruction or serosal involvement identified.

CT scan revealed endophytic soft tissue mass lesion is seen centered at the anterior gastric wall, measuring 6.1 × 5.2 × 3.8 cm in three orthogonal planes and existing homogenous enhancement in post contrast study with small areas of central breakdown however no extra-serosal extension. Consequent mild luminal encroachment with no significant gastric outlet obstruction. No suspicious lymph node enlargement. No calcification was observed with the abdominal CT (Figure 1). 

### 3.2. Endoscopic Findings

**Figure 2:**
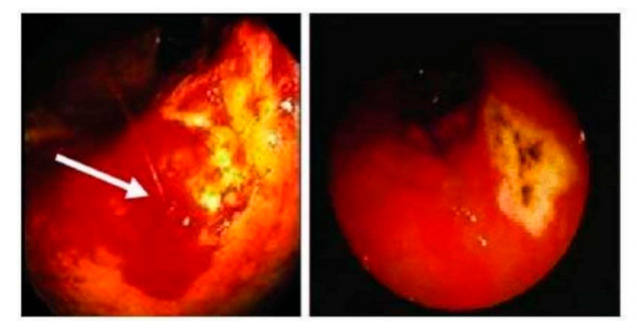
Endoscopic pictures showing ulcerated exophytic area at greater curvature covered with blood.

The gastric cavity was coated with altered blood. A localized ulcerated mass measuring about 5 × 4 cm was found at the proximal part of the greater curvature. The mass covered with abnormal mucosa with areas of ulcerations and bleeding, the mucosa was friable and bleeded easily on biopsy. The stomach was filled with blood. Multiple biopsies were taken from the different areas of the lesion (edges and centers) for histopathological study. The patient recovered smoothly without endoscopic or sedation complications (Figure 2). Follow up after surgery did not indicate recurrence.

### 3.3. The Intra-Operative Findings

**Figure 3:**
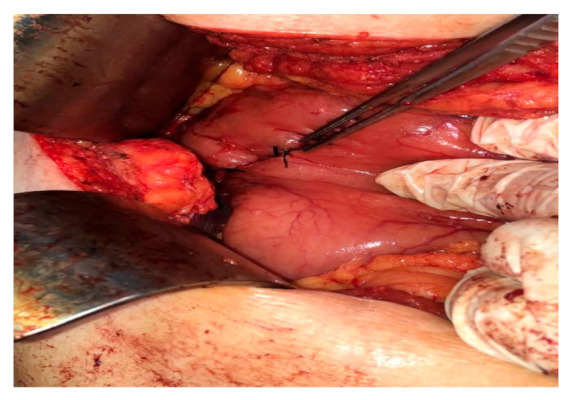
Intra-operative assessment. Image shows smooth serosal surface of stomach with no perforation.

Open examination of the stomach revealed local cicatrization of the anterior surface of the stomach near the lesser curvature with exophytic mobile intracavitary mass with focal erosion (Figure 3).

### 3.4. Gross Findings 

**Figure 4:**
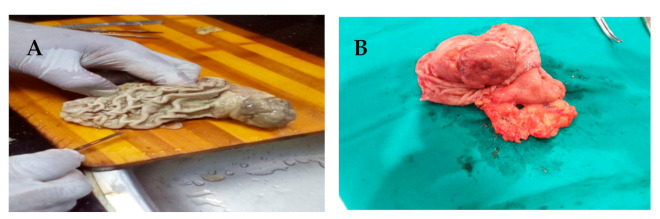
Gross picture of gastric piece showed polypoid and submucosal mass at the fundus with intact mucosal covering. (**A**) the lesion showed central erosion, piece of ometum tissue was seen attached to stomach part (**B**).

Partial gastrectomy for a specimen with omentum piece was done (the stomach measured 12 × 9 × 6 cm). On sectioning, it revealed polypoid grayish-white firm mass measured 7 × 5 × 4 cm with intact mucosa and focal erosions, with adequate surgical margins around (Figure 4A,B). A piece of unremarkable omental tissue was seen attached to the stomach part. No detected lymph nodes were found.

### 3.5. Histopathology Description and Immunohistochemistry

**Figure 5:**
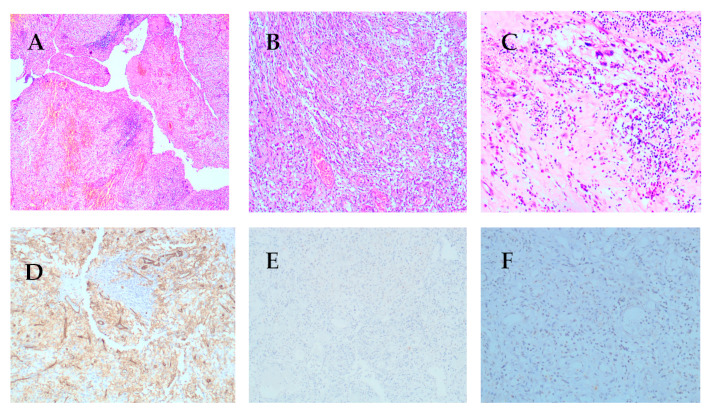
Common histological features of IFP. (**A**–**C**). (**A**) Submucosal spindle lesion intermixed with chronic inflammatory infiltrate and many dilated blood vessels (×100); (**B**) Spindle cell forming perivascular onion-skin arrangement (×200); (**C**) IFP with many eosinophils (×100); (**D**) CD34 strong and diffuse positivity of spindle cells. (**E**,**F**) CD117 & DOG-1 are negative for spindle cells. IFP: Inflammatory florid polyp.

**Figure 6:**
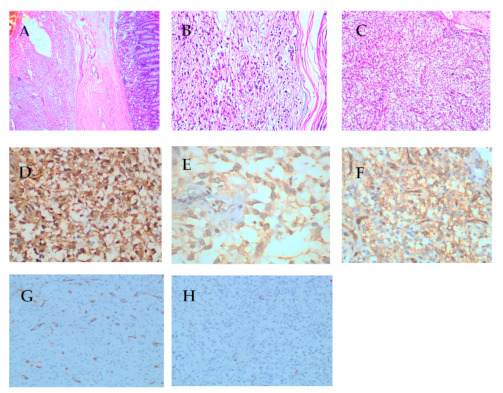
Common histological features of GISTs. (**A**–**C**). (**A**) Submucosal spindle cell neoplasm with intact mucosal covering (×100); (**B**) GIST with prominent nuclear palisading (×200); (**C**) GIST with mixed epitheloid and spindle neoplastic cell (×100); (**D**) DOG-1 strong and diffuse positivity of neoplastic cells. (**E**) CD117 with strong and diffuse positivity of neoplastic cells; (**F**) CD34 with strong and diffuse positivity of neoplastic cells. (**G**,**H**) SMA&S100 are negative for neoplastic cells.

Sections of the endoscopic biopsy showed very scanty bloody tissue and inconclusive for diagnostic assessment. Sections from the dissected mass revealed nodular neoplastic growth with intact mucosal covering and focal ulceration, there were two neoplastic lesions identified. The upper part showed submucosal lesion composed of spindle and stellate stromal cells in loose edematous stroma containing many thin-walled blood vessels with characteristic “onion skin” arrangement of spindle cells around the vessel, there is evidence of mixed inflammatory infiltrate rich in eosinophils, plasma cells, and lymphocytes with focal lymphoid aggregate formation (Figure 5A–C). The underlying tumor is composed of mixed spindle cells and epithelioid cells, most of them exhibited mild to moderate nuclear cytological atypia and mitosis (3/50HPF), Tumor cells extended through the muscularis propria (Figure 6A–C). Dissected surgical margins were negative for tumor cells.

Sections from the dissected polypoid mass showed that the tumor cells of the superficial area were negative for CD117, DOG1, Desmin, and S100 protein (Figure 5E,F) and showed strong positivity for CD34 (Figure 5D), these findings are consistent with IFPs. However the tumor cells in the deep part of the tumor were positive for CD117, DOG1 and CD34 (Figure 6D–F) and negative for S100 and Desmin (Figure 6G,H), these finding are consistent with GISTs.

## 4. Discussions

Collision tumors are defined as two histologically different adjacent neoplasms that do not intermingle. Collision tumors of the stomach, Collision tumors composed of GIST and other neoplasms have rarely been reported. To our best knowledge, there were less than 25 cases reported in the English literature [11]. 

The most common cases reported were gastric tumors involving GIST and adenocarcinoma [12]. The rare cases included; GIST with an inflammatory myofibroblastic tumor in a single gastric polypoid mass [13], gastric tumor involving the collision of GIST, angiosarcoma [8], and GIST with signet ring carcinoma [14]. GIST is the most common primary mesenchymal tumor of stomach [15]. It is usually positive for DOG-1 and CD117 (c-KIT), phenotypically had Cajal-cell differentiation, and in most cases contains CD117- or platelet-derived growth factor receptor alpha (PDGFRA)-activating mutations [16]. Approximately 60–70% of GISTs arise in the stomach. Rarely, GISTs may coexist with different types of cancer, either synchronously or metachronously [17]. 

IFP is a very rare entity that arises within the submucosa of the gastrointestinal tract [18]. The most common location of IFP is gastric antrum with presenting symptoms of epigastric pain and bleeding. The characteristic histologic features include perivascular onion skinning of spindle cells admixed with chronic inflammatory cells with many eosinophilic infiltrations [8].

Recently, evidence has shown that IFP is driven by an activating mutation in the *PDGFRA gene*, suggesting a neoplastic etiology [8]. The differential diagnosis of gastric IPF includes GIST, inflammatory myofibroblastic tumor, neural tumors, smooth muscle tumors and schwannoma [19]. 

The current case is the first example describing a collision tumor involving gastric GIST and IFP. Therefore, our case is the first case of a collision tumor containing a GIST and an IFP. As collision tumors are a rare process, the difficulty of the diagnosis and treatment is complex. It’s unlikely for clinicians and radiologists to expect a collision tumor initially. As in our case, neither the radiological report nor endoscopic assessment of the tumor gave any suggestion to the possibility of a collision tumor [14]. The histological findings of our case showed two different interface neoplasms, the first is GIST that composed of fascicles of spindle and epitheloid cells with rounded vesicular nuclei with pale eosinophilic cytoplasm, occasional mitotic figures 3/50HPF. Immunohistochemically, the neoplastic spindle cells of GIST are positive for CD117, DOG-1 [20], and CD34 and negative for S100 and SMA while CD117 and DOG-1 are negative for spindle cells of IFP. The GIST is interfaced superficially with IPF that is composed of spindle cell lesions admixed with chronic inflammatory cells with abundant eosinophils and many dilated blood vessels. The spindle cells are strongly positive for CD34 and negative for CD117 and DOG-1.

In our case, the diagnosis of GIST was made based on histopathology in combination with positivity for CD117 and DOG-1. The GIST was diagnosed as benign with low-risk potential due to the low mitotic count, size and to a lesser extent the low cellularity and mild atypia.

## 5. Conclusions 

We reported a case of a collision tumor consisting of a GIST and an IFP arising in the body of the stomach. This case is unique and the first report of a gastric collision tumor consisting of a GIST and an IFP. The final diagnosis has been based on the careful review of the clinical, radiological, histopathological, and immunohistochemical features of the tumor. Further investigation of the relationship between tumors of these types is needed.

## Data Availability

Data are available from the authors upon request.
